# Early Deafened, Late Implanted Cochlear Implant Users Appreciate Music More Than and Identify Music as Well as Postlingual Users

**DOI:** 10.3389/fnins.2019.01050

**Published:** 2019-10-11

**Authors:** Christina Fuller, Deniz Başkent, Rolien Free

**Affiliations:** ^1^Department of Otorhinolaryngology, Head and Neck Surgery, University Medical Center Groningen, University of Groningen, Groningen, Netherlands; ^2^Research School of Behavioral and Cognitive Neurosciences, Graduate School of Medical Sciences, University of Groningen, Groningen, Netherlands; ^3^Department of Otorhinolaryngology, Treant Zorggroep, Emmen, Netherlands

**Keywords:** early-deafened, late-implanted, cochlear implant, melody, postlingually deafened, implant outcome

## Abstract

**Introduction:** Typical cochlear implant (CI) users, namely postlingually deafened and implanted, report to not enjoy listening to music, and find it difficult to perceive music. Another group of CI users, the early-deafened (during language acquisition) and late-implanted (after a long period of auditory deprivation; EDLI), report a higher music appreciation, but is this related to a better music perception?

**Materials and Methods:** Sixteen EDLI and fifteen postlingually deafened (control group) CI users participated in the study. The inclusion criteria for EDLI were: severe or profound hearing loss onset before the age of 6 years, implantation after the age of 16 years, and CI experience more than 1 year. Subjectively, music perception and appreciation was evaluated using the Dutch Musical Background Questionnaire. Behaviorally, music perception was measured with melodic contour identification (MCI), using two instruments (piano and organ), each tested with and without a masking contour. Semitone distance between successive tones of the target varied from 1 to 3 semitones.

**Results:** Subjectively, the EDLI group reported to appreciate music more than postlingually deafened CI users. Behaviorally, while clinical phoneme recognition test score on average was lower in the EDLI group, melodic contour identification did not significantly differ between the two groups. There was, however, an effect of instrument and masker for both groups; the piano was the best-recognized instrument, and for both instruments, the masker with non-overlapping pitch was best recognized.

**Discussion:** EDLI group reported higher appreciation of music than postlingual control group, even though behaviorally measured music perception did not differ significantly between the two groups. Both surprising findings since EDLI CI users would be expected to have lower outcomes based on the early deafness onset, long duration of auditory deprivation, and on average lower clinical speech scores. Perhaps, the music perception difficulty comes from similar electric hearing limitations in both groups. The higher subjective appreciation in EDLI might be due to the lack of a musical memory, with no ability to compare music heard via the CI to acoustic music perception. Overall, our findings support a benefit from implantation for a positive music experience in EDLI CI users.

## Introduction

Music is an important daily-life auditory signal that can directly impact emotions, and also often plays an essential role in social entertainment and interactions (Boucher and Bryden, 1997; Drennan and Rubinstein, 2008; Salimpoor et al., 2009; Patel, 2014). The perception of the music signal, rich in acoustic cues, is unfortunately still challenging for users of cochlear implants (CIs) (e.g., McDermott, 2004; McDermott and Oxenham, 2008; Limb and Roy, 2014). In transmission of acoustic signals to the auditory nerve via electric hearing of the implant, due to the limitations of electric stimulation, the signal transmitted via CI is reduced to slow-varying envelopes delivered at a limited spectral resolution, whereby most fine cues needed for optimal music perception are lost (McDermott, 2004; Limb and Roy, 2014; Başkent et al., 2016). Another limiting factor for music perception is perhaps the functioning of the central and peripheral auditory pathway. Within the population of CI users, individuals have different neuronal survival (number of spiral ganglion cells) and/or morphological changes of nerve fibers (e.g., demyelination of the neuron soma of the spiral ganglion cells) (Nadol et al., 2001; Gassner et al., 2005; Seyyedi et al., 2014), due to different etiologies, age, and different periods of auditory deprivation (Teoh et al., 2004; Fallon et al., 2008; Peterson et al., 2010). These factors result in CI users having difficulties perceiving the richness of music, and especially of pitch and timbre (McDermott, 2004; McDermott and Oxenham, 2008; Limb and Roy, 2014), due to the limitations of the electric stimulation, combined with overall state of health of their auditory pathways.

Many music perception studies with CIs have been conducted with the typical implant user: a postlingually deafened (meaning, deafened after language acquisition) person that is implanted later in life. The overall outcomes show that, with the exception of rhythm identification, all other aspects of music perception (pitch, timbre and melody) are poorer in CI users than in normal hearing listeners, and that listening to music with the implant is also subjectively reported to be unsatisfying (Gfeller et al., 2000, 2002; Leal et al., 2003; McDermott, 2004; Galvin et al., 2007; Lassaletta et al., 2007, 2008b; Looi et al., 2012; Limb and Roy, 2014). Compared to hearing aid listeners CI users perform worse for music perception, apart from rhythm recognition (Looi et al., 2008). In case of a combined electrical and acoustical stimulation, frequency discrimination was better in hybrid listeners, instrument identification, and detection as bad between hybrid and CI only listeners (Brockmeier et al., 2010), whereas for real world music excerpts the hybrid and NH listeners outperform the CI user (Gfeller et al., 2006). In this study, we focus on a relatively new and less typical group of CI users; the early-deafened, late-implanted (EDLI) CI user. EDLI CI users are deafened during language acquisition (defined as deafness onset between 0 and 6 years in this study, based on literature), and only implanted after a longer period of auditory deprivation (implantation at or older than 16 years of age in this study, translating to at least 10 years of auditory deprivation) (Goorhuis-Brouwer and Schaerlaekens, 2000; van Dijkhuizen et al., 2011; Fuller et al., 2013; Heywood et al., 2016). The perception and appreciation of music in this EDLI CI group is mostly unknown. Yet, surprisingly, Fuller et al. (2013) showed that EDLI implant users reported higher appreciation of music compared to postlingually deafened CI users. More specifically, EDLI participants indicated that music sounds pleasant with a CI and rated the perceived quality of music higher than the postlingually deafened CI users did.

One reason for the discrepancy in subjectively reported music appreciation between the EDLI group and typical CI users might be that EDLI implantees rate music better due to a lack of an acoustical musical memory to compare the degraded signal of electric stimulation to, in contrast to postlingual CI users who often report music to sound worse than what they were used to before implantation (Mirza et al., 2003; Limb and Roy, 2014). Another difference between EDLI CI users and postlingually deafened CI users is the different development of auditory pathways and additionally the longer period of auditory deprivation at a young age in the early-deafened individuals. EDLI CI users have developed hearing loss during childhood, defined approximately between 1 and 6 years of age (Waltzman and Cohen, 1999; Sharma et al., 2002; Waltzman et al., 2002; Fallon et al., 2008; Peterson et al., 2010; Gordon et al., 2013), a timeframe during which the brain is best capable of speech and language development. If an individual develops hearing loss during this period a different shaping of the pathways for speech and language processing, as well as for music perception, might occur. Visual language development might interact with the auditory cortex (Champoux et al., 2009; Sandmann et al., 2012). Further the auditory cortex might not effectively process acoustical speech input (Teoh et al., 2004; Lazard et al., 2010). Due to this different development of the auditory pathways, some studies suggest that implantation might not be beneficial in this group, at least not for speech perception (Connell and Balkany, 2006; Medina et al., 2017). In fact, in clinical practice, up until recently, early-deafened individuals were not frequently implanted because of the expected low benefit of implantation for speech outcomes (Heywood et al., 2016). Next to the different neuronal network development in EDLI CI users, the long period of auditory deprivation (in our study at least 10 years) might also be influencing the outcome of implantation (Lazard et al., 2012; Blamey et al., 2013). In postlingually deafened and implanted individuals, the effect of auditory deprivation shows a negative influence on post-implantation speech perception (Lazard et al., 2012; Blamey et al., 2013). Literature suggests that speech perception is even poorer in the EDLI group than in the postlingually deafened group (Teoh et al., 2004; van Dijkhuizen et al., 2011). Some studies suggest to not implant individuals if they have had a 10 years or longer period of auditory deprivation (Connell and Balkany, 2006). Surprisingly, other studies show good implantation results with very low numbers of spiral ganglion cells (Blamey, 1997; Khan et al., 2005, Nadol et al., 2001), while others showed that a higher number of spiral ganglion cells are related to better speech recognition scores post-mortem (Seyyedi et al., 2014). Recently EDLI candidates have been implanted more, for example in the United Kingdom (Heywood et al., 2016). While results for speech perception remain—in general- lower than in postlingually deafened CI users, overall outcomes are still promising as this group often report improvements in quality of life as a result of implantation (Teoh et al., 2004; Santarelli et al., 2008; van Dijkhuizen et al., 2011; Fuller et al., 2013; Heywood et al., 2016).

To comprehensively investigate music perception and appreciation in EDLI CI users, in this study, we used both subjective and psychophysical measures. We investigated the subjective appreciation and perception of music and the psychophysical perception of music using melodic contour identification in EDLI implant users, in comparison to the control group of typical postlingual CI users. The research questions were: (1) Can we replicate our finding that EDLI CI users show a higher subjective music appreciation?; (2) If so, is the subjective music appreciation linked to a better psychophysical music perception?; (3) Are these subjective and psychophysical outcomes correlated, to investigate the potential relevance of the two measures to each other.

## Materials and Methods

### Participants

Sixteen EDLI CI users, as the test group (age range 23–75 years; seven female; demographic details presented in Table 1), and fifteen postlingually deafened CI users, as the control group (age range 48–75 years; five female; demographic details in Table 2), participated in the study. Four EDLI users overlapped with the earlier study by Fuller et al. (2013), but otherwise the test population differed between the two studies. All participants were native Dutch speakers and had 1 year or more CI experience. We aimed the two groups to be age- and gender-matched as much as possible, but despite this effort age still remained a significant factor [*F*_(1,31)_ = 27.99, *p* < 0.001], with EDLI participants being significantly younger than the control group. The inclusion criteria for EDLI were based on previous literature (Goorhuis-Brouwer and Schaerlaekens, 2000; Sharma et al., 2002; Connell and Balkany, 2006; van Dijkhuizen et al., 2011, 2016; Fuller et al., 2013; Heywood et al., 2016):

1. Severe or profound hearing loss onset before the age of six,
2. Implanted after the age of 16 years.

**Table 1:** Participant details of the EDLI CI users.

**Participant number**	**Age (years)**	**Onset of hearing loss (years)**	**Hearing aid use since (years)**	**Language**	**SIR**	**CI use (years)**	**Etiology**	**Clinical speech score (%)**
1	72	3	5	Dutch	5	10	Genetic	67
2	38	0	3	Frisian/Dutch	5	12	LVAS	85
3	41	0	0	Dutch/Frisian	3	2	Pendred Syndrome	95
4	64	0	6	Dutch	5	7	Unknown	82
5	62	5	5	Dutch	5	2	Meningitis	63
6	46	4	6	Dutch	5	16	Unknown	81
7	65	0	6	Dutch	5	13	Maternal rubella	90
8	67	0	4	Dutch	4	4	Meningitis	64
9	67	1	1	Dutch	4	5	Meningitis	40
10	62	0	<6	Sign language	3	6	Unknown	30
11	75	1	59	Dutch	5	5	Meningitis	45
12	55	0	4	Dutch with sign	3	1.5	Maternal rubella	60
13	23	0	3	Dutch with sign	4	7.5	Unknown	72
14	58	4	9	Dutch	4	7	Maternal rubella	69
15	62	0	3	Dutch	5	5	Asfyxia	85
16	63	4	21	Dutch	5	15	Unknown	78

**Table 2:** Participant details of the postlingually deafened CI users.

**Participant number**	**Age (years)**	**Onset of hearing loss (years)**	**Hearing aid use since (years)**	**Language**	**SIR**	**CI use (years)**	**Etiology**	**Clinical speech score (%)**
1	68	18	40	Dutch	5	5	Genetic	67
2	49	39	39	Dutch/Frisian	5	4	Trauma	90
3	69	61	61	Dutch/Frisian	5	7	Sudden deafness	75
4	69	46	46	Dutch	5	7	Ménière disease	90
5	69	32	32	Dutch	5	6	Trauma	93
6	68	50	50	Dutch	5	3	Genetic	88
7	49	34	34	Dutch	5	3	Genetic	90
8	74	31	31	Dutch	5	3	Unknown	69
9	66	18	18	Dutch	5	3	Genetic	79
10	65	55	57	Dutch/Frisian	5	2	Genetic	72
11	66	40	40	Dutch	5	2	Unknown	69
12	48	>18	27	Dutch	5	6	Labyrinth dysp.	93
13	66	45	42	Dutch	5	4	Unknown	78
14	75	33	39	Dutch	5	13	Unknown	78
15	74	50	50	Dutch	5	9	Otosclerosis	100

The inclusion criteria for postlingually deafened control CI users were:

1. Severe hearing loss onset after the age of 18, in order to ensure no overlapping period of early deafness with the EDLI participants.

The age of hearing loss onset was defined based on two sources, namely, the information the participants provided and their medical records. An important factor to note here is that all EDLI users implanted at our clinic were selected for implantation according to a special clinical protocol that is based on a speech intelligibility rating (SIR) (Samar and Metz, 1988) of the implant candidate's speech production, which has been shown to be an influencing factor on speech perception outcome (van Dijkhuizen et al., 2011). A score of three or higher (1–5 scale) indicates implantation candidacy; a score of 3 meaning: “Speech is difficult to understand; however the gist of the content can be understood.”; and 5 meaning: “Speech is completely intelligible.” The speech intelligibility is judged by an experienced speech therapist in our clinical team, and coupled to an expected outcome of implantation for speech. The clinicians use three outcome measures: (1) sound perception, no or minimal improvement of speech perception; (2) support of speech perception, some improvement in speech perception; (3) improved speech perception. Due to an expected lower implantation outcome, the patients scoring a SIR score 2 or lower receive a negative advise for implantation, which means that the person does not receive the CI. Apart from the language development aspect, EDLI users are additionally advised based on the audiological CI criteria, as well as the amount of auditory stimulation, intrinsic motivation, and medical history. Therefore, the selection of EDLI participants in this study might be biased towards relatively high implantation outcomes due to this specific selection procedure.

As a further characterization of our test and control groups of CI users we have also extracted clinical speech perception scores from the medical records. The test used in our clinic is based on recognition of phonemes in meaningful Dutch consonant-vowel-consonant words from the Nederlandse Vereniging van Audiologie (NVA) corpus, developed by Bosman and Smoorenburg (1995). During regular clinical visits, a list of 12 words spoken by a female speaker is presented at 75 dBA in free field in a sound-treated audiology booth. The 75 dBA level of loudness, a level representative of “loud” speech, was chosen here because a score for all users from both groups was available from our clinical database. The phoneme correct score of the last 11 words is calculated per visit per CI user. From these scores measured post-implantation, we selected the last known score from the clinic, as this would most realistically reflect speech perception performance of the participant around the time of this study. Note that the speech perception scores were not part of inclusion criteria in the present study, and were only used as a characterization of participants. And also worth noting that while these are clinical speech perception scores they are tested by different audiologists in different clinical booths during regular clinical outpatient visits, hence, some variation in these scores is expected also due to such external and circumstantial factors. The timing of these tests was at least 1 year after implantation.

**Figure 1:**
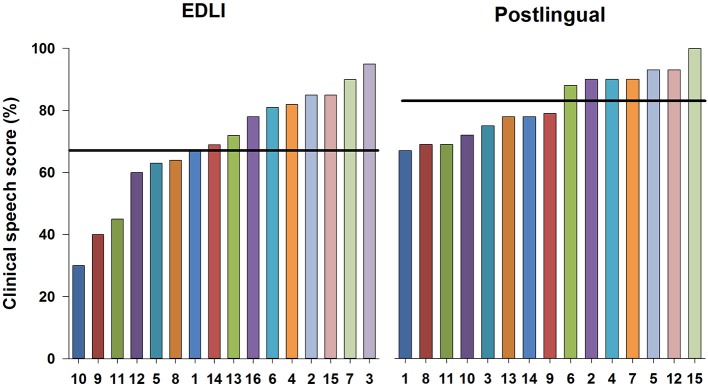
The clinical speech scores shown per individual for the EDLI participant group on the left and for the postlingually deafened control group on the right. The scores are arranged from lowest to highest score in each panel, from left to right. The numbers on the x-axis represent the individuals as numbered in Tables 1, 2. The horizontal line represents the mean score per group.

Figure 1 shows the outcomes for the latest clinical speech scores per group. The mean score for EDLI participants was 69%, for postlingually deafened CI users 82%. In the EDLI group a wider range in scores is observed (30–95%), whereas in the postlingual participant group a more consistent higher score range is observed (67–100%). A *t*-test showed a significant difference between the clinical speech scores of the two groups [*F*_(1,29)_ = −2.38; *p* = 0.02].

The Medical Ethical Committee of the University Medical Center Groningen (UMCG) reviewed and approved the research protocol. Before data collection started, all participants were given detailed information about study protocols. All participants provided written informed consent in accordance with the Declaration of Helsinki. Financial reimbursement based on the protocol of the Otorhinolaryngology Department of UMCG was provided for participation.

### General Procedures

There were two parts of the study. Subjective music appreciation was assessed via a questionnaire, which also included questions on satisfaction and listening habits. Music perception was assessed via a psychophysical test, namely melodic contour identification.

#### Dutch Musical Background Questionnaire

The Dutch Musical Background Questionnaire (DMBQ) is a translated (to Dutch) and edited version of the Iowa Musical Background Questionnaire (Gfeller et al., 2000). The questionnaire has three parts that measure: satisfaction with listening to music, self-perceived quality of music, and self-reported perception of the elements of music.

In this study, we chose three outcome measures from within these parts of the questionnaire:

1. Self-perceived quality of music,
2. Satisfaction with listening to music,
3. Music listening habits.

We chose these outcome measures: first to be able to compare the outcomes with those of Fuller et al. (2013); second since these outcomes represent the subjective music appreciation and enjoyment, the second most important outcome factor after speech as reported by CI users (Gfeller et al., 2000; Drennan and Rubinstein, 2008; Philips et al., 2012).

Participants, following informed consent, filled the questionnaires via a website digitally either at home or on a laptop at the outpatient clinic. The total time to fill the questions was about 10 min. Thirteen EDLI participants and 11 post-lingual CI participants filled the questions.

##### Self-perceived quality of music

The self-perceived quality of music is an indication of how music sounds under the best conditions with a CI. Visual analog scales (VASs) are used for 14 opposite adjective descriptors (unpleasant-pleasant, mechanical-natural, fuzzy-clear, does not sound like music-sounds like music, complex-simple, difficult to follow-easy to follow, dislike very much-like very much). The 10 centimeter scales range from 0 (negative quality) to 10 (positive quality). In this study, an average across the seven scales was taken to quantify the self-perceived quality of music.

##### Satisfaction with listening to music

The satisfaction of listening to music after implantation was measured using one item. The question was: Indicate which statement best describes how your enjoyment of listening to music has or has not changed after implantation. CI users could score three different outcomes:

1. Little or no satisfaction with listening to music,
2. The sound of music is okay or improving over time,
3. Music sounds pleasant.

The items were then scored on a scale of 0 (no satisfaction) to 2 (most satisfaction).

##### Music listening habits

Habits for listening to music were compared between before (retrospectively) and after implantation in both groups. Two questions were used:

1. I would describe myself as a person who often chooses to listen to music.
  Respondents indicated their agreement with the statement on a rating scale of one (“strongly disagree”) to four (“strongly agree”).
2. How many hours per week do you listen to music?
  This was scored on a rating scale of one to four: one = 0–2 h, two = 3–5 h, three = 6–8 h, and four = more than 9 h.

By adding these two items, one score before and one score after implantation were calculated for music listening habits. The total score, thus, could range from 2 (min. music listening) to 8 (max. music listening).

#### Melodic Contour Identification

The Melodic Contour Identification (MCI) test was originally developed by Galvin et al. (2007) and used multiple times in CI studies (Galvin et al., 2007, 2008, 2009, 2012; Fuller et al., 2014, 2018), measuring the identification of nine different melodic contours. By using different instruments the effect of timbre, and by using a masker contour the effect of melody masker can be investigated. The MCI test in this study was configured as in Fuller et al. (2014) to be able to compare the outcomes of our present study with our former study that measured MCI in postlingually deafened CI users. The test consisted of five-tone melodic contours with a total of nine different pitch directions: “Rising,” “Flat,” “Falling,” “Flat-Rising,” “Falling-Rising,” “Rising-Flat,” “Falling-Flat,” “Rising-Falling,” “Flat-Falling”). 220 Hz was the lowest note per contour. A 1, 2, or 3 semitone distance between the successive notes in the contours was used. Each note was 250 ms long, and the silent interval between notes was 50 ms. Two instruments were used: piano and organ, as in Galvin et al. (2008). MCI was measured with and without a competing contour, the “masker.” The masker was always the “flat” contour played by the piano (Galvin et al., 2009). The maskers differed in pitch: a pitch [A3 (220 Hz)] overlapping with target, and another pitch [A5 (880 Hz)] non-overlapping with target. A total of six conditions were tested: (1) piano target alone (no masker), (2) piano target with the A3 piano masker, (3) piano target with the A5 piano masker, (4) organ target alone (no masker), (5) organ target with the A3 piano masker, and 6) organ target with the A5 piano masker. Both masker and target started at the same time, meaning the notes of both were played at the same time.

##### Melodic contour identification setup

The psychophysical test MCI was conducted in an anechoic chamber at UMCG. Participants were asked to set their CI to their normal daily modus and volume. This setting was not changed during testing. In case of a bimodal participant, they were asked to remove the hearing aid from the contra-lateral ear during testing. CI users were seated facing a single speaker (Tannoy Precision 8D; Tannoy Ltd., UK) at one-meter distance. The stimuli were presented using MATLAB 2016a (The Mathworks, inc., USA) implemented on a Mac computer (MacOS, El Capitain; Apple, California, USA) and via an Audiofire 4 Audio Recording Interface with preamplification (Echo Audio Corporation, California, USA) and a DA10 digital-to-analog converter (Lavry Engineering Inc.). The stimuli were presented at 65 dB SPL, indicating an audible and comfortable, daily level of loudness. Furthermore, this level is in line with former studies, making a fair comparison possible. After an update of testing room, the stimuli were presented using an Apple Mac mini (MacOS, High Sierra; Apple, California, USA), MATLAB 2018a (The Mathworks, inc., USA), and a MOTU UltraLite-mk4 soundcard.

##### Melodic contour identification procedure

The contours were visually depicted on a touchscreen monitor [GPEG AOD (Advantech, USA)], which was placed 1 m in front of the participant. After listening to the audio contours, the participants directly indicated via the touchscreen the matching visual contour on the screen, and the results were stored immediately via MATLAB. During training, the participants identified one round of nine contours using three repetitions, a total of 27 contours per instrument without the masker, during which visual feedback was provided. The order of testing during data collection was: piano without a masker, then with the piano with A3, followed by the piano with the A5 masker. After the three piano conditions the same procedure was followed for the organ. During data collection, the nine contours were presented in random order, and were repeated three times per round, thus 27 contours per round. A total of 6 × 27 = 162 contours were played in the testing phase, during which no feedback was provided. A percentage correctly identified contours was calculated per condition by the MATLAB software automatically. The total testing time was 30 min.

##### Statistics

IBM SPSS Statistics 23 was used for the statistical analysis. *T*-tests defined the differences between the groups for the outcomes of the questionnaires. Split-plot repeated measures analysis of variance (ANOVA) was used to analyse the differences between the EDLI group and the postlingually deafened CI users for MCI with a Greenhouser-Geisser correction. Within subject factors were presence of masker (masker, no masker) and instrument (piano, organ). For the satisfaction with listening to music comparison between both groups a Chi-square test with a Monte Carlo simulation was run. For listening habits a Kruskal-Wallis test was computed. Two-tailed Pearson correlations were conducted between the subjective and psychophysical outcomes. A *p* < 0.05 was considered significant.

## Results

### Subjective—Dutch Musical Background Questionnaire

#### Self-Perceived Quality of Music

**Figure 2:**
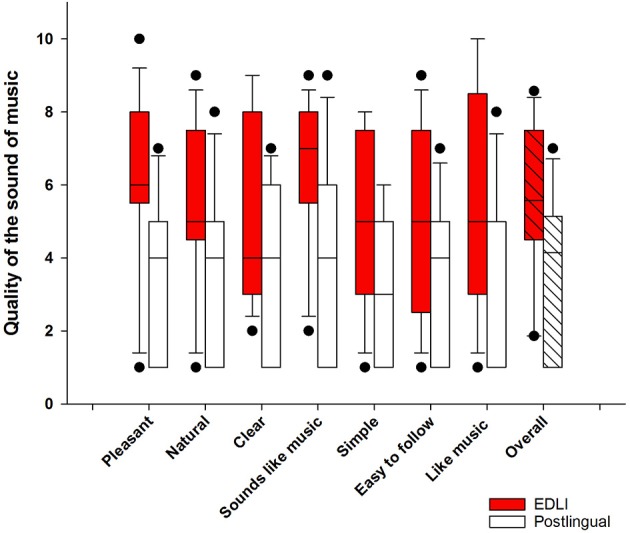
The self-perceived quality of music for EDLI (in red) and postlingually deafened CI users (in white). The boxes represent the 25–75 percentile, the lines the median values, and the error bars the 10–90 percentile. The dots indicate the outliers.

Figure 2 shows the results for the self-perceived quality of music for the EDLI group (in red) and postlingually deafened CI users (in white). Results show a higher perceived quality of the sound of music for EDLI CI users compared to postlingually deafened CI users. To calculate whether there is a difference in overall quality of music, we averaged over all seven scales to create an overall quality of music outcome score (boxes most on the right side in Figure 2). An analysis of variance (ANOVA) with group as a between-subjects factor and the quality of music as within-subjects factor was performed. A significant main effect for group was shown [*F*_(1,22)_ = 6.41, *p* = 0.02]. No significant effect was shown for the quality of music or the interaction between group and quality of music. It should be noted that not all participants filled the questionnaires. For the EDLI group 13 participants filled the questionnaire, in the control group 11.

#### Satisfaction With Listening to Music

Table 3 shows the percentages of both groups for the satisfaction with listening to music after implantation. Notable is the difference in distribution between the two groups, as almost all postlingually deafened CI users are in the second category and none in the last, while the EDLI CI users are diffuse across all three categories. Further, 23% of EDLI CI participants reported music to sound pleasant while no postlingual CI participant reported music to sound pleasant. A Chi-square test with a Monte Carlo simulation was run that showed a significant difference in the distribution between the groups *X*^2^ (2, *N* = 24 = 5.67, *p* = 0.049).

**Table 3:** The percentages of both groups for the satisfaction with listening to music after implantation.

	**EDLI (*n* = 13)**	**Postlingual (*n* = 11)**
Little or no satisfaction with listening to music	31% (*n* = 4)	8% (*n* = 1)
The sound of music is okay or improving over time	46% (*n* = 6)	92% (*n* = 10)
Music sounds pleasant	23% (*n* = 3)	0% (*n* = 0)

#### Music Listening Habits

**Figure 3:**
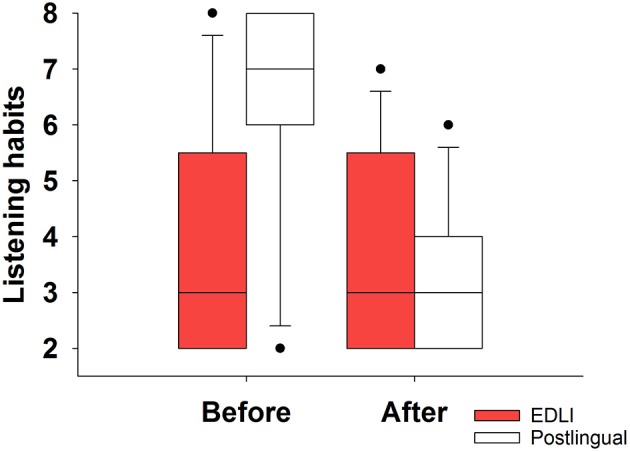
The music listening habits before and after implantation for EDLI and postlingually deafened CI users. The box descriptions are similar to those of Figure 2.

Figure 3 shows the self-reported music listening habits before and after implantation for EDLI CI users and postlingual CI users. Postlingually deafened CI users reported to listen to music more before implantation compared to the EDLI participants, but they reported their listening habits drop after implantation. A Kruskal Wallis test was performed to compare the listening habits before and after implantation between the groups. A significant difference was shown before implantation (1, *N* = 24 = 8.22, *p* = 0.04) and no difference after implantation (1, *N* = 24 = 0.035, *p* = 0.85).

### Psychophysical—Melodic Contour Identification

**Figure 4:**
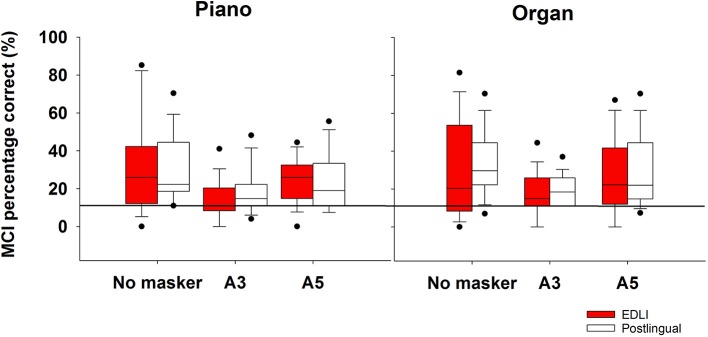
The average percentage correct scores for the melodic contour identification for the piano (left) and organ (right) shown for both groups. From left to right in each panel the masker is shown: no masker, A3 overlapping pitch masker, A5 non-overlapping pitch masker. The box descriptions are similar to those of Figure 2. The thick horizontal line represents chance level.

Figure 4 shows the results for the MCI test for piano and organ (left and right panels, respectively). The three different conditions from left to right are: no masker, A3 overlapping pitch masker, A5 non-overlapping pitch masker. Performance was worst for the overlapping pitch condition for both instruments in both groups. A split-plot repeated measure analysis of variance (RM ANOVA) with group (two levels; EDLI, postlingually deafened) as between-subjects factor, and instrument (two levels; piano, organ) and masker (three levels; no masker, A3 masker, and A5 masker) as within-subjects factors was performed. The complete analysis is shown in Table 4. There were main significant effects for instrument [*F*_(1,29)_ = 6.03; *p* < 0.02], with the organ being the best recognized instrument, and masker [*F*_(1.61,46.57)_ = 14.25; *p* < 0.001], with the A3 masker being the most difficult condition. No significant main effect was found for group [*F*_(1,29)_ = 0.10; *p* = 0.76]. No significant interactions were observed. The observed power was low for the group effect, indicating that larger participant groups would be needed for a potential difference between the two groups' performance.

**Table 4:** The results of the split-plot repeated measures ANOVA for MCI.

**Between-subjects factor**		**Observed power**
Group	*F*_(1,29)_ = 0.10; *p* = 0.76	0.06
**Within-subjects factors**		
Instrument	*F*_(1,29)_ = 6.03; *p* < 0.02^*^	0.66
Masker	*F*_(1.61,46.57)_ = 14.25; *p* < 0.001^*^	0.99
Instrument x Masker	*F*_(1.89,54.68)_ = 0.26; *p* = 0.72	0.01
Instrument x Group	*F*_(1,48)_ = 0.45; *p* = 0.51	0.10
Masker x Group	*F*_(1.61,46.57)_ = 0.26; *p* = 0.72	0.09
Instrument x Masker x Group	*F*_(1.89,54.68)_ = 1.01; *p* = 0.37	0.21

* *significant (p < 0.05)*.

### Correlations Between Subjective and Psychophysical Measures

A correlational analysis using Spearmans correlation test (two-tailed) was performed between the overall reported music quality and the outcomes of the MCI and the clinical speech scores for both groups (see Table 5). No systematic correlations were found. For EDLI participants, a significant positive correlation between the quality of music and the piano A3 masker was shown, but none with the clinical speech scores. For the postlingually deafened participants, no correlations were shown between the subjective and psychophysical measures.

**Table 5:** Correlational analyses between the subjective outcomes of the quality of music clinical speech scores, and the psychophysical MCI outcomes for both piano and organ, shown for EDLI and control group separately (upper and lower parts, respectively).

**EDLI**	**Quality of music**	**Clinical speech scores**
Piano	*r* = 0.574	*r* = 0.240
	*p* = 0.07	*p* = 0.48
	*N* = 11	*N* = 11
Piano A3 masker	*r* = 0.683	*r* = −0.04
	*p* = 0.02^*^	*p* = 0.90
	*N* = 11	*N* = 11
Piano A5 masker	*r* = 0.447	*r* = 0.349
	*p* = 0.17	*p* = 0.29
	*N* = 11	*N* = 11
O*r*gan	*r* = 0.480	*r* = 0.321
	*p* = 0.14	*p* = 0.34
	*N* = 11	*N* = 11
Organ A3 masker	*r* = 0.453	*r* = 0.244
	*p* = 0.16	*p* = 0.47
	*N* = 11	*N* = 11
Organ A5 masker	*r* = 0.455	*r* = 0.304
	*p* = 0.16	*p* = 0.36
	*N* = 11	*N* = 11
Clinical speech scores	*r* = −0.033	X
	*p* = 0.92	X
	*N* = 13	X
***Postlingual***		
Piano	*r* = −0.333	*r* = 0.191
	*p* = 0.32	*p* = 0.57
	*N* = 11	*N* = 11
Piano A3 masker	*r* = 0.148	*r* = 0.317
	*p* = 0.66	*p* = 0.34
	*N* = 11	*N* = 11
Piano A5 masker	*r* = 0.181	*r* = 0.234
	*p* = 0.59	*p* = 0.49
	*N* = 11	*N* = 11
Organ	*r* = −0.131	*r* = 0.027
	*p* = 0.70	*p* = 0.94
	*N* = 11	*N* = 11
Organ A3 masker	*r* = 0.037	*r* = 0.092
	*p* = 0.91	*p* = 0.79
	*N* = 11	*N* = 11
Organ A5 masker	*r* = 0.143	r = 0.119
	*p* = 0.68	*p* = 0.73
	*N* = 11	*N* = 11
Clinical speech scores	*r* = −0.084	X
	*p* = 0.81	X
	*N* = 11	X

* *significant*.

## Discussion

The purpose of the study was to investigate the subjective music appreciation and psychophysical music perception in EDLI CI users and to compare these results to the more typical group of postlingually deafened CI users. Most of previous research with EDLI CI users had been done on speech perception. Even though implantation outcome or speech perception were not part of inclusion criteria in the present study, our observations based on clinical speech scores using phoneme-in-word identification were in line with this previous literature on EDLI implant users; our test group of EDLI users perform lower on the clinical speech perception test than our postlingually deafened control CI users and the test group shows a wider variation in outcomes. These differences likely indicate the effects from long-term auditory deprivation in EDLI participants. Based on the lower speech outcomes found in our study group and reported in literature (Teoh et al., 2004; Santarelli et al., 2008; van Dijkhuizen et al., 2011), we expected the EDLI participants to have lower performance in melody identification as well, especially since music is considered an even more complex acoustical signal than speech (McDermott, 2004; McDermott and Oxenham, 2008; Limb and Roy, 2014). Despite this expectation based on previous literature, Fuller et al. (2013) did, surprisingly, show that EDLI CI users subjectively report to enjoy listening to music more than postlingually deafened participants report. Consistently with our former study, EDLI participants of the present study also subjectively reported a higher quality of music than postlingually deafened CI users. There was no difference between the two groups in listening habits after implantation, whereas postlingually deafened CI users reported to listen to music before implantation significantly more than EDLI CI users. Interestingly, EDLI CI users reported to be more satisfied with listening to music after implantation compared to postlingual CI users. For the psychophysical test of music perception, namely, the melodic contour identification, against our expectation, EDLI implant users performed as well as postlingually deafened CI users. EDLI participants thus scored the perceived quality of music higher, and reported to be more satisfied with listening to music than postlingual CI participants, even though the psychophysical music outcomes for MCI were comparable between the groups.

### Subjective Results—Dutch Musical Background Questionnaire

Psychophysically both groups performed the same for music perception, but subjectively, surprisingly, EDLI users judged the overall, subjective quality of music significantly higher than postlingually deafened CI users. Thus, our clinical speech outcomes, which were significantly lower for EDLI group than that of the postlingual control group, confirmed our group to be an EDLI group that fits with the lower expected outcomes from literature. In contrary to this expectation, it is a surprising finding that the performance does not differ for melody identification, and it is even more surprising that subjectively EDLI users appreciate music more than postlingual users. This finding is, however, in line with our previous results reported in Fuller et al. (2013) that also showed a high subjective appreciation of music. In the current study, we included only four EDLI participants that overlapped with our previous study, hence the present results successfully replicated our former results with a relatively new EDLI test population. The positive subjective music appreciation shown by Fuller et al. (2013) and replicated in our study, hence, provide strong evidence that EDLI have a more positive experience of music with the implant.

Not only the quality of music is more positively judged by the ELDI group, but there was also a higher satisfaction with listening to music with the implant. Compared to our former study the EDLI participants of this study are less satisfied; while in this study 23% ticked the “music sounds pleasant” box 60% did so in the previous study. This is however still more positive than the postlingually deafened participants, since none of them indicated that music sounds pleasant. Supporting the latter, other studies have also shown multiple times that the typical CI user is unsatisfied with listening to music (Gfeller et al., 2000; Lassaletta et al., 2008b; Looi and She, 2010).

One might argue that these differences are due to a lack of musical memory from acoustic hearing in EDLI CI users, and that therefore they judge the quality of music to a different scale than postlingually deafened CI users that likely use acoustical music memory as an anchor for their judgments of music quality with an implant (Galvin et al., 2009; Limb and Roy, 2014). Two observations from this study give support to this idea. Firstly, while postlingual CI users reported a large drop in their music listening habits from pre- to post-implantation, EDLI participants reported no change in music listening from pre- to post-implantation. Hence, it is possible that this relative negative change in music listening in postlingual group is an indication of less appreciation of music post-implantation in comparison to pre-implantation acoustic hearing, while EDLI show no such effect. Secondly, that there were no systematic correlations between subjective music appreciation measures and psychophysically measured MCI scores perhaps also indicate that what is subjectively reported relies more on psychological factors, instead of actual perceptual performance with music. On the other hand, alternatively, another influencing factor might be specific to our test population at UMCG, due to our clinical implantation protocol at our implant center in Groningen. The clinical protocol calls for a strict selection of implant candidates and only the EDLI candidates with best SIR scores are implanted. As a result, potentially some of our EDLI participants might have had some acoustic input with a (likely power) hearing aid, as vibrations of low frequencies, or some hearing with very loud music. Of course, given the level of hearing loss reported by participants or in the medical charts for this group, likely the quality of music via these means was different than that with a CI. Most postlingually deafened CI users definitely had a longer period of usable acoustical hearing and thus richer musical experience than the EDLI participants. For clinical speech scores, however, we did find a difference between the two groups, showing the EDLI perform worse for speech perception, perhaps due to a different language acquisition experience during childhood, and in line with what literature suggests. For music perception, however, there might be another explanation. Perhaps the auditory pathways that are involved in the processing of music in the brain: (1) for the actual perception for music; and (2) for the rewarding system related to music appreciation (Blood et al., 1999; Blood and Zatorre, 2001; Peretz et al., 2001), might have been developed differently in EDLI individuals. The limbic and reward regions can cause an emotional response to listening to music, which can be related to familiarity of the musical excerpt (Pereira et al., 2011). If familiarity combined with a musical memory plays a role in the emotional, subjective reaction to music, this might be one of the factors contributing to the difference in music appreciation between the EDLI group and the postlingually deafened participants. Since EDLI CI users have a non-existing or minimal acoustical musical memory, the emotional reaction is supposedly not largely driven by the familiarity from the acoustical memory, but only by the musical memory developed with the CI. Furthermore, this rewarding system might have only been built with the CI, causing only familiarity with listening to music with the CI. Together these experiences might create a more positive response in EDLI participants than postlingually deafened participants for listening to music with a CI.

#### Listening Habits

The self-reported post-implantation listening habits did show a difference between the two groups of CI users. For postlingually deafened CI users, a decline in listening habits after implantation was shown, as was previously found in various studies (Gfeller et al., 2000; Mirza et al., 2003; Lassaletta et al., 2008a; Migirov et al., 2009; Looi and She, 2010; Philips et al., 2012). This decrease is probably caused by the different experience of listening to music via electrical hearing post-implantation compared to acoustical hearing pre-implantation in this group. There was no significant difference in reported listening habits between the two groups after implantation. Since there was no difference between the two groups in post-implantation listening habits one might argue that there might be other reasons, apart from quality of the sound, or the satisfaction, that cause the (relatively) small amount of time spent on listening to music with a CI. Perhaps listening to music is just very effortful with a CI for all CI users. Since we know the complexity of music makes CI users to perform lower on music identification compared to NH listeners (McDermott and Oxenham, 2008; Limb and Roy, 2014), subjectively CI users also report to prefer less complex music categories compared to NH listeners (Veekmans et al., 2009). Perhaps music is, as is speech in noise perception (Cullington and Zeng, 2011), such an effortful task for CI users, something CI users cannot simply afford to do for many hours a day. A last argument might be that EDLI CI users, since they were suffering from (severe) hearing loss for most of their lives, still do not listen often to music with the implant, as it has not been a large part of their daily life anyway and therefore they simply listen as much as they do before implantation. Last, the EDLI participants were younger then the postlingually deafened CI users and might for this reason appreciate music more, as was shown by Mirza et al. (2003) who showed that younger CI users enjoy music more. Concluding, EDLI CI users show a more positive appreciation of music than postlingually deafened CI users, yet, no differences for listening habits with the implant have been found.

### Psychophysical—Melodic Contour Identification

Based on the poorer average implantation outcomes in EDLI CI users for clinical speech perception scores, we expected our EDLI group to also perceive music less well than postlingually deafened CI users. Surprisingly, however, the EDLI and postlingually deafened CI users performed evenly on the melody identification test. The piano was the most difficult instrument to recognize, and the A3 masker with overlapping ground note the most difficult condition for both instruments. The results for the postlingually deafened CI users are in line with former studies (Galvin et al., 2007, 2008, 2009; Fuller et al., 2018). The performance in CI users is lower than in NH listeners that score for all conditions with a mean ranging from 71 (for piano A3) to 80% (for organ without a masker) (Fuller et al., 2014). One interpretation for the lack of difference in performance between the groups might be that the task does not depend on former acoustical, musical input over many years. Some basic pitch perception ability seems to be already developed early in human life. For example, it has been shown in 5 to 11 months old infants that they are capable of distinguishing differing pitch contours, most likely based on sensitivity to temporal cues (Jusczyk and Krumhansl, 1993; Trehub and Hannon, 2006). A well-known example is children preferably listen to child-directed speech, which has exaggerated pitch contours, over adult-directed speech (Fernald and Kuhl, 1987). Given the MCI task stimuli were basic MIDI melodies with no temporal, spectral, vocal, instrumental etc. complexity, perhaps EDLI CI users can sufficiently rely on such an early-developed ability when performing the MCI task with the CI, as do postlingually deafened CI users.

Another reason might be that perhaps the MCI task is independent of the period of auditory deprivation and a different auditory pathway. Perhaps the MCI task measures the ability of the naïve CI user for detection of a semitone sequence; or the MCI captures the real limitations of electric hearing, and does not depend on the differences in auditory pathways, auditory deprivation, or age of deafness. This, however, is contradicted by the finding that MCI performance can be trained in CI users (Galvin et al., 2007, 2012; Patel, 2014; Fuller et al., 2018). One might therefore expect a postlingually deafened participant to have a better auditory pathway for semitone detection than an EDLI CI user. Perhaps in future studies different materials that use more complex melodies, real life music excerpts, or using a range of musical instruments, would show a difference between the groups.

### Correlations

To investigate whether the subjective and psychophysical outcomes in our study were associated, and if potential musical appreciation judgment was based on how well a CI user does with music perception, we correlated the subjective measures with the psychophysical measures for melody identification and clinical phoneme identification for both groups. We ran multiple tests, which only showed one significant correlation, something that might be caused by multiple testing, or might be caused by the size of our groups, causing our study to be possibly underpowered. Therefore, we conclude that no systematic correlations were shown. Even though differences were shown between the subjective judgment of both groups, and no differences between the psychophysical behavior, hence, no correlation was shown between the subjective measures and the psychophysical measures.

For speech perception, based on clinical speech scores, also, no correlations with MCI performance or music appreciation were shown for both groups. For the EDLI group this is in line with our former study, where also no correlation was shown between speech perception and music appreciation, but what is newly shown here is the finding that this also does not correlate with MCI performance. Since MCI only measures one aspect of music perception, melody recognition, and it does not measure any other aspects of music, perhaps it is difficult to relate to more real-life outcomes as speech perception and subjective music appreciation, which cover larger aspects of these auditory domains. For postlingually deafened CI users, however, some studies had indicated music perception to be correlated to speech perception. Galvin et al. (2007) specifically showed a correlation between vowel recognition and MCI performance. In their study, the range of semitones used, however, was wider (1–5) and therefore overall performance for MCI was higher, compared to our study where we only used a 1–3 semitone distance, the most difficult test settings. Additionally, our clinical speech perception test is not a vowel test, but a phoneme recognition task. It is possible that identification of an isolated vowel relies more on better decoding of phonetic sounds of speech while identifying phonemes embedded in meaningful words engage other and higher-level mechanisms of speech processing, where other linguistic cognitive factors, such as use of lexical knowledge and context, also play a role.

Another factor of influence is the lack of statistical power due to the limited number of participants in the study and the multiple tests that were run. This might give a biased result for the correlations. In future a higher number of participants is needed to draw a certain conclusion about the correlations between the behavioral and subjective data.

Still, the surprising lack of correlations might indicate that subjective judgment of music appreciation is not entirely determined by perception accuracy, as measured by psychophysical outcomes in both groups, indicating that appreciation is perhaps based on more psychological factors.

### Early-Deafened, Late-Implanted Cochlear Implant Users

A potential selection bias is present in our study, even though inclusion criteria for EDLI target group were well-defined based on literature, and average clinical speech score of EDLI group was significantly lower than the average score of the control group, again as would be expected from literature. The source of this potential bias is that EDLI candidates are selected and counseled by our implant team based on certain pre-implantation criteria. For example, based on van Dijkhuizen et al. (2011) who found that the SIR score is related to a better post-implantation speech perception outcome, only participants with a SIR-score 3 or higher are selected for implantation as part of the clinical procedure. A SIR-score 3 or higher indicates that the implant candidate's spontaneous speech is understandable, if necessary, that the listener (speech therapist) is concentrating and perhaps reading lips. Below the score 3, implantation candidate's speech is not understandable, apart from a few words or parts of words. This might have introduced some bias in our study, as our test group perhaps included individuals with the best intelligible speech production. We are aware from the clinical records that there are also EDLI implantees that do not use their CI due to a (either subjective or objective, or both) lack of implantation gain, and EDLI implantees that do not perform well for speech outcomes, but still use the device for sound perception/awareness only. None of these implantees volunteered for this study, leading to EDLI participants who had meaningful clinical speech scores (>30%). Therefore, the EDLI cases of no implant use or no measurable or minimal speech outcome are not represented within our EDLI test group of the present study.

Whether the selection of implantees based on speech scores might have an influence on the music perception and appreciation scores is unknown. An interesting follow-up question might be whether implantees with a SIR-score lower than two, would show the same perceptional and subjective outcomes (related to music) as implantees with a higher SIR-score.

Last, it should be noted that, despite our efforts of matching ages of the two groups, our EDLI users were younger in general than the postlingually deafened control group. Age might therefore be an influencing factor on the outcomes of our study, as a younger age was shown to potentially contribute to higher and better speech and music perception outcomes with a CI in postlingually deafened participants (Sladen and Zappler, 2015).

All in all, the outcomes of this study support implantation in selected early deafened individuals, even after a (relatively long) duration of auditory deprivation. Potential benefits for implantation in EDLI group were supported by our study for music perception, both when subjectively and psychophysically measured. Many of our EDLI CI users, while as a group on average lower in clinical test scores than postlingual CI users, still showed relatively good speech perception benefit, and all EDLI participants had measurable clinical test scores. Hence, in addition to potential gain in speech understanding benefits from implantation, the comparable MCI performance, and the higher subjective judgment of music in EDLI participants indicate additional positive potential outcomes of implantation in this group.

## Data Availability Statement

The dataset is available via: https://hdl.handle.net/10411/QIWMKJ.

## Ethics Statement

The studies involving human participants were reviewed and approved by Medical Ethical Committee of the University Medical Center Groningen. The patients/participants provided their written informed consent to participate in this study. Written informed consent was obtained from the individual(s) for the publication of any potentially identifiable images or data included in this article.

## Author Contributions

CF performed the data analysis and wrote the first draft of the manuscript. All authors contributed to manuscript revision, read, approved the submitted version, contributed to conception, and design of the study.

### Conflict of Interest

The authors declare that the research was conducted in the absence of any commercial or financial relationships that could be construed as a potential conflict of interest.
